# Ureteral Injury with Delayed Massive Hematuria after Transvaginal Ultrasound-Guided Oocyte Retrieval

**DOI:** 10.1155/2015/760805

**Published:** 2015-06-04

**Authors:** Tanja Burnik Papler, Eda Vrtačnik Bokal, Vesna Šalamun, Dejan Galič, Tomaž Smrkolj, Nina Jančar

**Affiliations:** ^1^Division of Obstetrics and Gynecology, Department of Human Reproduction, University Medical Centre Ljubljana, Šlajmerjeva 3, SI-1000 Ljubljana, Slovenia; ^2^Division of Surgery, Department of Urology, University Medical Centre Ljubljana, Zaloška 7, SI-1000 Ljubljana, Slovenia

## Abstract

We report a case of ureteral injury with delayed hematuria after transvaginal oocyte retrieval. A 28-year-old infertile patient with a history of previous laparoscopic resection of endometriotic nodes of both sacrouterine ligaments presented with abdominal pain one day after oocyte retrieval. Four days after oocyte retrieval, she presented with massive hematuria that reappeared 6 days after oocyte retrieval. Monopolar coagulation with wire electrode and insertion of a double-J-stent was performed during operative cystoscopy. The patient recovered completely after transfusion and had no signs of renal impairment after ureteric stent removal. This is the first report of ureteral injury after oocyte retrieval presenting itself with delayed massive hematuria and no signs of renal dysfunction or urinary leakage into retroperitoneal space.

## 1. Introduction

Transvaginal ultrasound-guided oocyte retrieval is a standard method in women undergoing IVF procedures. The method was described by Wikland et al. [1], and it is preferred to laparoscopic or transabdominal oocyte retrieval, since it is less invasive. Nevertheless, cases of bleeding [2], infection [3], and injuries of the adjacent organs [4] have been described after this procedure.

We report for the first time a case of ureteral injury that presented with delayed massive hematuria without urinary tract signs of peritoneal irritation 4 and 6 days after ultrasound-guided transvaginal oocyte retrieval.

## 2. Case Presentation

A twenty-eight-year-old nulliparous woman with a 2-year history of infertility underwent her first IVF attempt at our tertiary infertility centre in January 2014. Her hormonal status (LH, FSH, estradiol, and prolactin), hysterosalpingography, and partner's sperm parameters were within the normal range. Four months prior to this IVF attempt she underwent a hysteroscopy and laparoscopy. Resection of uterine septum, electrocoagulation of peritoneal endometriotic lesions, and resection of endometriotic nodes of both sacrouterine ligaments were performed during these endoscopic procedures. There was no note of an abnormal course of ureters at that time.

Controlled ovarian hyperstimulation was achieved by administering 150 IU of recombinant FSH follitropin beta (Puregon, NV Organon, Oss, Netherlands) combined with GnRH antagonist cetrorelix acetate (Cetrotide, Asta Medica, Frankfurt, Germany). After 8 days of stimulation, 10000 IU of human chorionic gonadotropin (Pregnyl, NV Organon, Oss, Netherlands) was administered for oocyte maturation. Transvaginal ultrasound-guided oocyte retrieval was performed 36 hours later. Six oocytes were retrieved and she was discharged after 1 hour of uncomplicated postoperative course.

One day after oocyte retrieval she presented to the emergency department complaining of lower abdominal pain. Her temperature, pulse, and blood pressure were within the normal range. Laboratory analysis revealed mild anemia (hemoglobin 112 g/L) and a slightly elevated C-reactive protein (14 mg/L), whereas white blood cell count and electrolytes were within the normal range. Serum urea and creatinine levels were normal. There was no muscular guarding during abdominal palpation; however, there was bilateral adnexal tenderness. There was a trace of blood in the vagina upon examination. Except for the enlarged, multicystic ovaries and a small collection of anechogenic fluid in the pouch of Douglas, there were no abnormalities found on transvaginal ultrasound scan at the admission. She was admitted for observation and pain management. She received crystalloid infusions, intravenous analgesics, and oral iron therapy. In the following days, she was asymptomatic, hemodynamically stable, without any tenderness of the abdomen upon palpation. The control vaginal ultrasound showed the presence of hematoma measuring 5 centimeters behind the uterus on the left side. The collection of fluid in the pouch of Douglas did not enlarge. She was discharged on the fourth day after oocyte retrieval.

Later that day she was admitted again, complaining of bloody urine. A total of 1300 mL of bloody urine was collected with bladder catheterization. Laboratory analysis revealed further anemia (hemoglobin level 95 g/L) and low blood sodium (127 mmol/L). Cystoscopy performed by urologist revealed normal bladder mucosa and efflux of bloody urine from the left ureteral orifice but found no active bleeding. Indwelling catheter (Ch18) was inserted, and the urine was clear thereafter. On day 5, she had 3 embryos: 2 blastocysts and 1 arrested embryo. Her clinical status was considered to be compatible with ET, since she was hemodynamically stable without any subjective complaints. One blastocyst was transferred and one blastocyst was cryopreserved. The pregnancy test 14 days after oocyte retrieval was positive; however, empty gestational sac was seen on ultrasound examination 4 weeks after ET.

Two days later, six days after oocyte retrieval, a massive hematuria reappeared, and there was a further worsening of anemia, with hemoglobin level of 73 g/L. She received 2 units of concentrated erythrocytes. Abdominal ultrasound revealed approximately 500 mL of free fluid or blood in the abdominal cavity. There was approximately 500 mL of blood and blood clots in the bladder. There was no free fluid or pathological formations in the retroperitoneal space. The kidneys were normal; pyelocaliceal system was not dilated. Cystoscopy was repeated, blood clots were rinsed from the bladder, and active bleeding was seen from the left ureteral orifice. Therefore, she was transferred to the operating theatre for endoscopic hemostasis in general anesthesia. Active bleeding from the left ureter was observed and left sided ureteroscopy using semirigid ureterorenoscope showed active bleeding from the intramural part of the left ureter, approximately 1 cm above the orifice. Hemostasis was achieved using monopolar coagulation with wire electrode, and double-J-stent was inserted into the left ureter. Antibiotic prophylaxis with gentamycin 160 mg intravenously was administered during the procedure. She received another 3 units of concentrated erythrocytes and 2 units of fresh frozen plasma. On the next day, hemoglobin level was 113 g/L. Control abdominal ultrasound revealed an empty bladder, without the presence of blood clots; double-J-stent and catheter balloon were seen in the bladder. Approximately 500 mL of free fluid was seen, surrounding the spleen, in the hepatorenal pouch, and surrounding the right ovary. There were no collections of fluid in the retroperitoneal space, around the great vessels and psoas muscles. Renal sonogram was also normal. The patient was asymptomatic and urine culture was negative. The urine catheter was removed 7 days after her second cystoscopy and she was discharged in stable condition. Double-J-stent was removed 6 weeks later. Control abdominal ultrasound was within the normal range and the patient had no consequences of ureteral injury.

## 3. Discussion

Despite the ureter's anatomical location (anterolaterally to the upper fornices of the vagina), very few cases of clinically recognizable ureteral injury after transvaginal oocyte retrieval have been reported [5–11]. The reported ureteral injuries resulted in ureteric obstruction and hydronephrosis [5, 8, 11], ureteric transection or stricture requiring surgical reimplantation [6, 7], urine leakage into retroperitoneal space [9], ureterovaginal fistula [6, 10], and, in the most severe case with delayed diagnosis, unilateral kidney failure requiring nephrectomy [5]. To the best of our knowledge, no case of massive hemorrhage following ureteric injury after oocyte retrieval has been reported so far.

Among the factors that increase the risk of ureteral injury are the presence of pelvic inflammatory disease, previous pelvic surgery, endometriosis, and the presence of pelvic adhesions. These conditions can potentially alter the anatomic course of the ureter. In two previous reports of ureteral injury after transvaginal oocyte retrieval [5, 7], the injury happened in patients with known endometriosis, pelvic adhesions, and repeated ovarian punctures. Our patient had also previously undergone laparoscopic bilateral resection of sacrouterine ligaments due to the deep infiltrating endometriosis. Scar formation after the resection of endometriotic nodes could have shortened sacrouterine ligaments and thus changed the anatomical course of ureters. For this reason, ureters of our patient could have been more prone to injury, despite the fact that normal ureteral course was seen during the initial laparoscopy.

This is the first case report of severe delayed hematuria and hemorrhagic shock after ureteral injury. The injury was diagnosed 6 days after oocyte retrieval. Clinicians dealing with our patient 4 days after oocyte retrieval were convinced that hematuria resulted from urinary bladder injury that occurred during transvaginal oocyte retrieval. Catheter insertion would have been sufficient therapy for the management of extraperitoneal bladder injury [12]. Previously reported ureteral injuries presented themselves with lower abdominal, flank, or suprapubic pain [5–11], urinary symptoms such as dysuria, urinary urgency, vesical tenesmus, and microscopic or macroscopic hematuria [6, 9, 11], and gastrointestinal symptoms such as nausea and vomiting [6, 8]. The onset of symptoms in the reported cases varied from a couple of hours [8] to a couple of months [5]. In our patient, the presenting symptom was abdominal pain, which could have been present due to the enlarged ovaries following ovarian stimulation. Abdominal pain could also be the sign of bleeding into abdominal cavity after oocyte retrieval but the ultrasound findings were within a normal range for a patient who underwent oocyte retrieval after controlled ovarian hyperstimulation. There was a presence of free fluid in the pouch of Douglas that was estimated to be less than 200 mL in volume upon the admission, and this amount is considered to be normal after oocyte retrieval [13, 14]. However, in the IVF patient, it is difficult to optimally evaluate pelvic organs using ultrasound, since there are changes in ovarian volume and structure as a result of ovarian stimulation and follicular aspiration. Injury of the adjacent organs, such as ureter or urinary bladder, following oocyte retrieval could also present itself with abdominal pain. However, there were no urinary symptoms or signs of peritoneal irritation such as nausea, vomiting, and muscular guarding in our patient. Also, there were no abnormalities in the laboratory analysis related to ureteral injury, since the values of serum urea and creatinine were not elevated. Additionally, there were no signs of urine leakage. For this reason, we did not suspect urinary tract injury before the appearance of a massive hematuria.

Due to this atypical clinical course, we presume that the ureter was not completely transected. Probably one of the vessels supplying the pelvic ureter was injured during the oocyte retrieval, resulting in a massive urinary and intraperitoneal hemorrhage. The distal ureter is supplied by the branches of the common iliac and internal iliac arteries, particularly uterine and superior vesical arteries [15]. We presume that one of the branches of uterine or vesical arteries had been injured in our patient during the oocyte retrieval procedure, thus causing the hematoma behind the vaginal wall on the left side, with delayed intraperitoneal bleeding and hematuria. We speculate that the hematoma closed the injured ureter and vessel in the first few days after oocyte retrieval. The delayed hematuria and intraperitoneal bleeding could have been due to the resorption and demarcation of the hematoma around the injured ureter and vessel. Moreover, stronger waves of peristaltic contractions of ureter after intensive hydration of our patient could have been responsible for reopening of injured ureteral wall. Ureteral injury in women with risk factors for altered ureteral course may be prevented by positioning the aspiration needle more laterally, in order to avoid the needle passage through the parametrial tissue containing the ureter and vessels.

Ureteral injury following oocyte retrieval is a rare but potentially serious complication that can compromise renal function, if it remains undetected. Differential diagnoses of this complication are pelvic infection, ovarian torsion, and intraovarian and intraperitoneal bleeding. Our patient had previously undergone laparoscopic surgical procedure with bilateral resection of endometriotic nodes of sacrouterine ligaments. This implies that the course of the ureter in our patient could have been affected due to the previous surgical procedure or the endometriosis itself. Positioning the needle more laterally during the oocyte retrieval could be a useful preventive measure in such cases. Furthermore, stenting of ureters prior to surgical procedure in order to increase their visibility has been proposed, as a preventative measure to avoid ureteral injury. Placing the ureteral stent prior to oocyte retrieval has been beneficial in a case report by Vilos et al. [16], resulting in uneventful oocyte retrieval in a patient with previous ureteral injury during transvaginal oocyte retrieval. However, gross hematuria and renal insufficiency have been reported to occur after ureteral stenting [17]; therefore, the benefits of this invasive procedure prior to transvaginal oocyte retrieval should outweigh the risks. Also, pelvic segment of ureters can be identified with the use of transvaginal ultrasound [18], so every physician performing transvaginal ultrasound-guided oocyte retrieval should try to identify them prior to and during the procedure.

In conclusion, suspicion of a potential ureteral injury should be raised with every patient presenting with the aforementioned symptoms after oocyte retrieval, especially in patients with known conditions that can affect normal pelvic anatomy.

## References

[1] Wikland M., Enk L., Hamberger L. (1985). Transvesical and transvaginal approaches for the aspiration of follicles by use of ultrasound. *Annals of the New York Academy of Sciences*.

[2] Evers J. L. H., Larsen J. F., Gnany G. G., Sieck U. V. (1988). Complications and problems in transvaginal sector scan-guided follicle aspiration. *Fertility and Sterility*.

[3] Dicker D., Dekel A., Orvieto R., Bar-Hava I., Feldberg D., Ben-Rafael Z. (1998). Ovarian abscess after ovum retrieval for in-vitro fertilization. *Human Reproduction*.

[4] Bergh T., Lundkvist O. (1992). Clinical complications during in-vitro fertilization treatment. *Human Reproduction*.

[5] Jones W. R., Haines C. J., Matthews C. D., Kirby C. A. (1989). Traumatic ureteric obstruction secondary to oocyte recovery for in vitro fertilization: a case report. *Journal of In Vitro Fertilization and Embryo Transfer*.

[6] Coroleu B., Lopez Mourelle F., Hereter L. (1997). Ureteral lesion secondary to vaginal ultrasound follicular puncture for oocyte recovery in in-vitro fertilization. *Human Reproduction*.

[7] Fugita O. E., Kavoussi L. (2001). Laparoscopic ureteral reimplantation for ureteral lesion secondary to transvaginal ultrasonography for oocyte retrieval. *Urology*.

[8] Miller P. B., Price T., Nichols J. E., Hill L. (2002). Acute ureteral obstruction following transvaginal oocyte retrieval for IVF. *Human Reproduction*.

[9] Fiori O., Cornet D., Darai E., Antoine J. M., Bazot M. (2006). Uro-retroperitoneum after ultrasound-guided transvaginal follicle puncture in an oocyte donor: a case report. *Human Reproduction*.

[10] von Eye Corleta H., Moretto M., D'Avila Â. M., Berger M. (2008). Immediate ureterovaginal fistula secondary to oocyte retrieval—a case report. *Fertility and Sterility*.

[11] Grynberg M., Berwanger A. L., Toledano M., Frydman R., Deffieux X., Fanchin R. (2011). Ureteral injury after transvaginal ultrasound-guided oocyte retrieval: a complication of in vitro fertilization-embryo transfer that may lurk undetected in women presenting with severe ovarian hyperstimulation syndrome. *Fertility and Sterility*.

[12] Gomez R. G., Ceballos L., Coburn M. (2004). Consensus statement on bladder injuries. *BJU International*.

[13] Dessole S., Rubattu G., Ambrosini G., Miele M., Nardelli G. B., Cherchi P. L. (2001). Blood loss following noncomplicated transvaginal oocyte retrieval for in vitro fertilization. *Fertility and Sterility*.

[14] Ragni G., Scarduelli C., Calanna G., Santi G., Benaglia L., Somigliana E. (2009). Blood loss during transvaginal oocyte retrieval. *Gynecologic and Obstetric Investigation*.

[15] Shafik A. (1972). A study of the arterial pattern of the normal ureter. *Journal of Urology*.

[16] Vilos A. G., Feyles V., Vilos G. A., Oraif A., Abdul-Jabbar H., Power N. (2015). Ureteric injury during transvaginal ultrasound guided oocyte retrieval. *Journal of Obstetrics and Gynaecology Canada*.

[17] Chahin F., Dwivedi A. J., Paramesh A. (2002). The implications of lighted ureteral stenting in laparoscopic colectomy. *Journal of the Society of Laparoendoscopic Surgeons*.

[18] Pateman K., Mavrelos D., Hoo W.-L., Holland T., Naftalin J., Jurkovic D. (2013). Visualization of ureters on standard gynecological transvaginal scan: a feasibility study. *Ultrasound in Obstetrics and Gynecology*.

